# Splenic Rupture Secondary to Amyloidosis: A Case Report and Review of the Literature

**DOI:** 10.3390/hematolrep15020038

**Published:** 2023-06-06

**Authors:** Hisham F. Bahmad, Samantha Gogola, Lorena Burton, Ferial Alloush, Mike Cusnir, Michael Schwartz, Lydia Howard, Vathany Sriganeshan

**Affiliations:** 1 The Arkadi M. Rywlin M.D. Department of Pathology and Laboratory Medicine, Mount Sinai Medical Center, Miami Beach, FL 33140, USA; 2 Herbert Wertheim College of Medicine, Florida International University, Miami, FL 33199, USA; 3 Department of Internal Medicine, Mount Sinai Medical Center, FL 33140, USA; 4 Department of Internal Medicine, Division of Hematology and Oncology, Mount Sinai Medical Center, Miami Beach, FL 33140, USA; 5 Department of Pathology, Herbert Wertheim College of Medicine, Florida International University, Miami, FL 33199, USA

**Keywords:** spleen, rupture, amyloid light chain, amyloidosis, plasma cell myeloma, case report, review

## Abstract

Amyloidosis is a term describing the extracellular deposit of fibrils composed of subunits of several different normal serum proteins in various tissues. Amyloid light chain (AL) amyloidosis contains fibrils that are composed of fragments of monoclonal light chains. Many different disorders and conditions can lead to spontaneous splenic rupture, including AL amyloidosis. We present a case of a 64-year-old woman with spontaneous splenic rupture and hemorrhage. A final diagnosis of systemic amyloidosis secondary to plasma cell myeloma was made with infiltrative cardiomyopathy and possible diastolic congestive heart failure exacerbation. We also provide a narrative review of all documented cases of splenic rupture associated with amyloidosis from the year 2000 until January 2023, along with the main clinical findings and management strategies.

## 1. Introduction

Amyloidosis is a term describing the extracellular deposit of fibrils composed of subunits of several different normal serum proteins in various tissues [1]. These fibrils can be easily identified via biopsy, due to the fact that they have a very characteristic appearance in electron microscopy, by their ability to bind Congo red, resulting in apple green birefringence under polarized light, and thioflavin T, producing yellow-green fluorescence [2]. 

Amyloid light chain (AL) amyloidosis, also referred to as immunoglobulin light chain amyloidosis, contains fibrils that are composed of fragments of monoclonal light chains [1,3]. Patients who are affected by AL amyloidosis may present with amyloidosis either alone or in conjunction with other plasma cell dyscrasias such as multiple myeloma (MM), Waldenstrom macroglobulinemia, and monoclonal gammopathy of undetermined significance (MGUS) [3]. All forms of systemic amyloidosis in which fibrils are derived from monoclonal light chains, regardless of the nature of the underlying plasma cell disorder, are considered AL amyloidosis [3].

Splenic rupture is a life-threatening condition leading rapid intra-abdominal blood loss that worsens rapidly and can lead to hypovolemic shock, and has a mortality rate of up to 10% [4]. Many different disorders and conditions can lead to spontaneous splenic rupture, including AL amyloidosis [4]. 

We present a case of a 64-year-old woman with spontaneous splenic rupture and hemorrhage. Pathology showed that the spleen was entirely replaced with amyloid deposits. A bone marrow biopsy was performed, yielding a diagnosis of plasma cell myeloma. This case report was conducted and reported in accordance with the Surgical CAse REports (SCARE) guidelines for reporting case reports. We also provide a narrative review of all documented cases of splenic rupture associated with amyloidosis from the year 2000 until January 2023, along with the main clinical findings and management strategies. 

## 2. Methods

We conducted a comprehensive review [5], beginning with a thorough search, using PubMed as the primary database, for mesh terms, keywords, and combinations as follows: ((spleen[MeSH Terms]) AND (rupture[MeSH Terms])) AND ((light chain amyloidosis[MeSH Terms]) OR (amyloid[MeSH Terms]) OR (amyloidosis[MeSH Terms]) OR (immunoglobulin amyloidosis[MeSH Terms]) OR (primary amyloidosis[MeSH Terms]) OR (multiple myeloma[MeSH Terms]) OR (waldenstrom macroglobulinemia[MeSH Terms]) OR (monoclonal gammopathy of undetermined significance[MeSH Terms])). 

**Figure 1:**
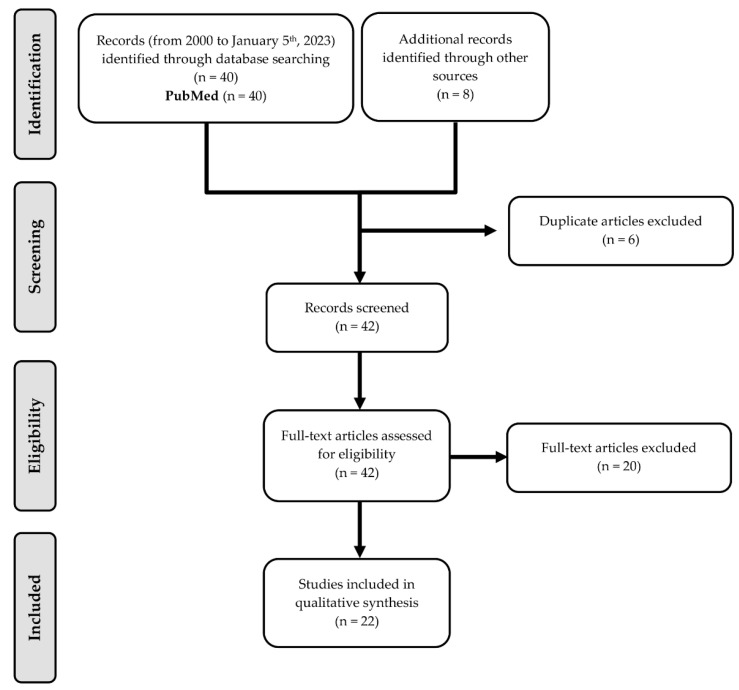
Flow diagram representing the review process.

All published articles from the year 2000 to until January 2023, were included. A total of 40 studies were retrieved using this search algorithm. All studies were assessed for eligibility, after which 20 were excluded based on their abstracts. Twenty articles were included for whole-text analysis. The literature was also searched during the same time frame using the mentioned MeSH terms. A total of 8 articles were retrieved, 6 of which overlapped with the prior search results. The remaining 2 articles were included for whole-text analysis. Of the 22 articles included for whole-text analysis, none were excluded based on their content (Figure 1).

## 3. Case Presentation

A 64-year-old woman with a history of gastroesophageal reflux disease, hypertension, and arrhythmia presented to our hospital for dull abdominal pain localized to the right upper quadrant with a one-day duration. The pain radiated to the right and left flanks and iliac region and was associated with nausea and vomiting. The patient denied cough, chest pain, or fever. Her surgical history was significant for hysterectomy and vesicovaginal fistula repair. No relevant family history was present. The patient had no known allergies.

Upon presentation, the patient’s vital signs were as follows: arterial blood pressure was 108/82 mmHg, pulse was 80 beats per minute, respiratory rate was 16 breaths per minute, temperature was 36.7 °C, and SpO_2_ was 99%. The patient’s physical examination was significant for non-specific and diffuse abdominal tenderness. The laboratory results are summarized in Table 1. The patient had mild leukocytosis and normocytic normochromic anemia. Her electrolyte levels were within normal limits. Blood urea nitrogen (BUN), serum creatinine, Troponin I, lactic acid, prothrombin time (PT), AP thromboplastin time (aPTT), international normalized ratio (INR), liver enzymes, and liver function test results were all within the normal reference ranges. Urinalysis showed 30 mg/dL protein in the urine. Urine and blood cultures were negative.

**Figure 2:**
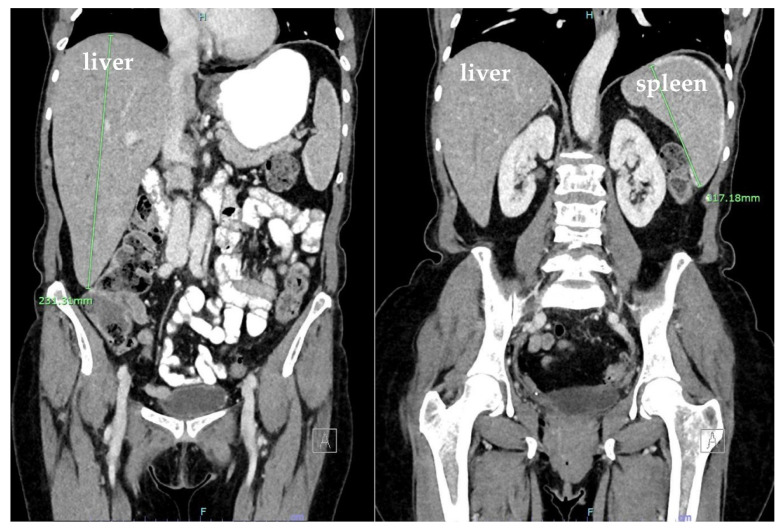
Coronal plane computed tomography (CT) scan with IV contrast of the abdomen and pelvis showing hepatomegaly (23 cm). Spleen was normal.

**Figure 3:**
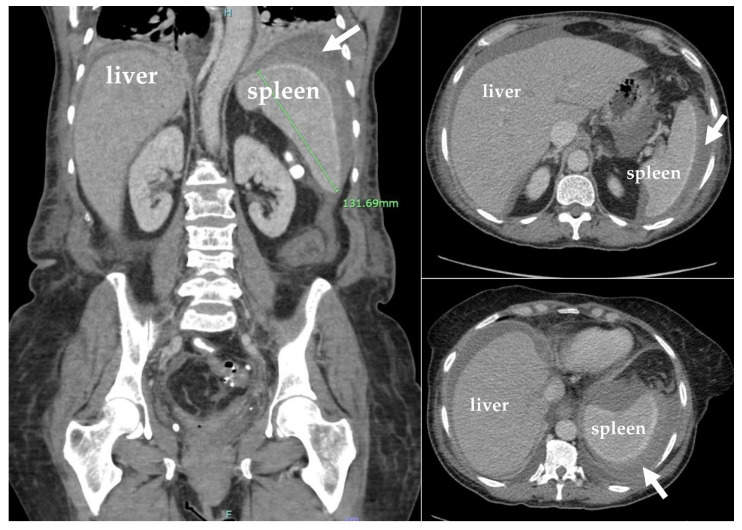
Coronal and sagittal plane computed tomography (CT) scans with IV contrast of the abdomen and pelvis showing hemoperitoneum (white arrows) and mild splenomegaly (13.2 cm).

Computed tomography (CT) of the abdomen and pelvis revealed hepatomegaly of 23 cm with no other acute findings (Figure 2). During hospitalization, hemoglobin dropped to 6.1 g/dL, requiring transfusion of a unit of packed RBC, which improved hemoglobin to 7.0 g/dL. The nausea and vomiting persisted, warranting a repeat CT of the abdomen and pelvis, which showed new development of hemoperitoneum in the left abdomen surrounding the spleen, leading to concerns about subcapsular hematoma and an enlarged spleen by 13.2 cm (Figure 3). Additionally, the patient developed dyspnea, and chest X-ray revealed a pleural effusion in the left lung base.

**Figure 4:**
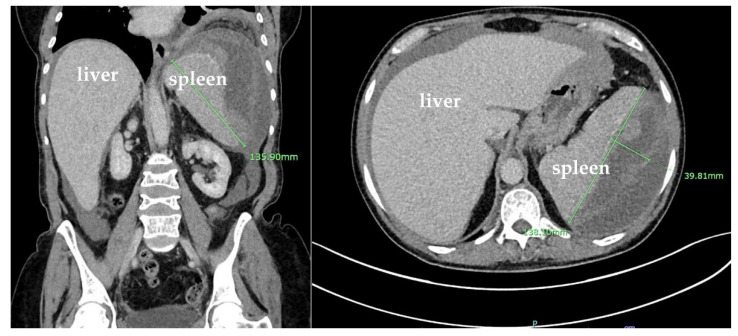
Coronal and sagittal plane computed tomography (CT) scans with IV contrast of the abdomen and pelvis showing large mixed-density splenic subcapsular hematoma of unclear etiology, which significantly increased in size since the prior study, this time measuring 13.8 × 13.6 × 4 cm (previously 13.2 cm in greatest dimension), with a mass effect on the splenic parenchyma and an increase in the moderate-volume hemoperitoneum.

The CT angiography (CTA) of the abdomen and pelvis was negative for active bleeding. The patient was discharged 9 days after the stabilization of anemia and the resolution of her nausea and vomiting. However, she returned to the hospital 15 days later due to a syncopal episode and persistence of her abdominal pain. During that admission, she was hypotensive and was found to have anemia with hemoglobin of 6.9 g/dL that required another transfusion of one unit of packed RBCs. Repeated CT of the abdomen and pelvis showed large mixed-density splenic subcapsular hematoma of unclear etiology, which had significantly increased in size since the prior study, this time measuring 13.6 × 13.8 × 4 cm (previously 12 × 11 × 0.1 cm), with a mass effect on the splenic parenchyma and an increase in the moderate-volume hemoperitoneum (Figure 4). 

The interventional radiology department performed angiography, which revealed no extravasation of the contrast, and also performed coil embolization of the proximal splenic artery to contain the bleeding. Proximal splenic artery embolization decreases the perfusion pressure in the spleen but allows for viability of the spleen to be maintained via collateral pathways [6]. The patient was discharged after her condition was stabilized. Five days later, she was readmitted for worsening abdominal pain and shortness of breath. Laboratory studies showed leukocytosis of 12.99 × 10^3^/μL, stable normocytic anemia with hemoglobin of 9.6 g/dL, and mild elevation of troponin at 56 pg/mL. Urinalysis showed an increase in urine protein to 100 mg/dL.

**Figure 5:**
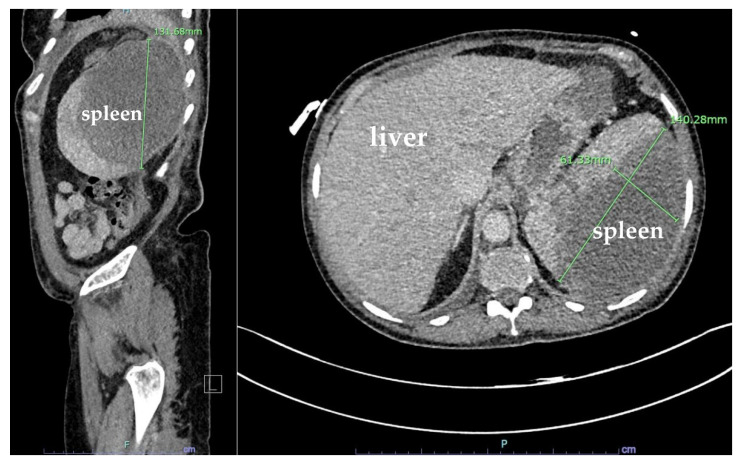
Coronal and sagittal plane computed tomography angiogram (CTA) scans with IV contrast of the abdomen and pelvis showing large splenic infarction with an increase in the size of splenic subcapsular hematoma, measuring up to approximately 14.0 × 13.2 × 6.1 cm, with a mass effect on the splenic parenchyma and an increase in the moderate-volume hemoperitoneum.

**Figure 6:**
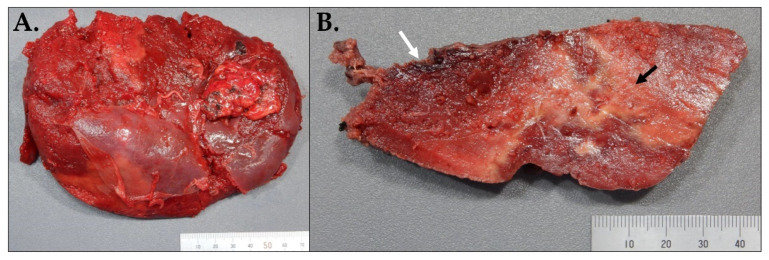
Gross images of the spleen. (**A**) The splenic capsule is dark red in color and has a predominantly posterior laceration which measures 13.5 cm. Multiple adhesions can be also noted. (**B**) Cut surface of the spleen shows a poorly demarcated, dark red, hemorrhagic area located superiorly in the subcapsular area and measuring 10.5 × 7 × 4 cm, and covered by a tan rind (white arrow). Multiple ill-defined, firm, white-yellow areas are also noted, comprising approximately 10% of the cut surface (black arrow).

A CTA of the abdomen and pelvis was performed and showed an increase in the size of the splenic subcapsular hematoma, measuring approximately 14.0 × 13.2 × 6.1 cm, and large splenic infarction (Figure 5). Given the new imaging findings and intractable left abdominal pain, the surgery team was consulted. Accordingly, the patient was taken to the operation room for open splenectomy due to splenic rupture and hemorrhage. The spleen was then taken to pathology. On histopathological examination, the spleen was enlarged, weighing 593 g and measuring 16 × 10 × 5.2 cm. The capsule was dark red and had a predominantly posterior laceration, which measured 13.5 cm. Multiple adhesions were also noted (Figure 6A). The cut section revealed a poorly demarcated, dark red, hemorrhagic area located superiorly in the subcapsular area and measuring 10.5 × 7 × 4 cm. Additionally, identified were multiple ill-defined, firm, white-yellow areas comprising approximately 10% of the total cut surface. The remainder of the spleen parenchyma was homogeneous, dark red, and firm (Figure 6B).

**Figure 7:**
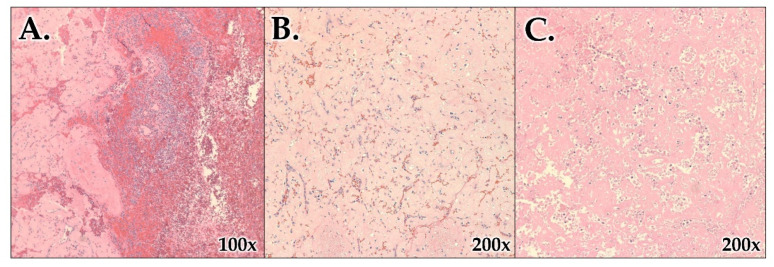
Microscopic images of the spleen. Areas of hemorrhage, inflammation, granulation tissue (**A**), necrosis and infarction (**B**), and eosinophilic amorphous deposits (**C**) are seen with no identifiable splenic parenchyma (i.e., white pulp, marginal zone, and red pulp) (H&E stain; objectives ×100 in (**A**) and ×200 in (**B**,**C**)).

On microscopic examination, areas of necrosis and infarction, hemorrhage, inflammation, granulation tissue, and deposits of eosinophilic amorphous material were appreciated. No identifiable splenic parenchyma (i.e., white pulp, marginal zone, and red pulp) was seen (Figure 7). Interestingly, Congo red-positive amyloid deposits were present, signifying the involvement of amyloidosis, AL (lambda)-type. Liquid chromatography–tandem mass spectrometry (LC MS/MS) was performed on peptides extracted from Congo red-positive, microdissected areas of the paraffin-embedded blocks, which detected a peptide profile consistent with AL (lambda)-type amyloid deposition. Tissue flow cytometry of the spleen failed to reveal a monoclonal B-cell population or an aberrant T-cell immunophenotype.

A transthoracic echocardiogram was performed, showing normal left ventricular systolic function with a left ventricular ejection fraction (LVEF) of 70%. The global longitudinal strain (GLS) was impaired, measuring −14.3% (normal < −16%) with relative apical sparing. There was also severe left ventricular hypertrophy (LVH) and indeterminate diastolic function consistent with infiltrative cardiomyopathy, which is highly suggestive of cardiac amyloidosis. 

Further laboratory tests revealed elevated pro-Brain Natriuretic Peptide (BNP) levels of 9971.0 pg/mL (reference range 10.0–300.0 pg/mL) and excess production of serum Lambda free light chains of 12,673.5 mg/L. Serum Kappa free light chains were normal at 11.8 mg/L. The free Kappa/Lambda ratio was therefore very low at <0.01. Excess production of free Kappa or Lambda chains can alter the ratio of free serum light chains. Serum protein electrophoresis with reflex IFX showed hypogammaglobulinemia with gamma globulin of 0.32 g/dL (reference range 0.71–1.54 g/dL), and serum immunofixation electrophoresis noted monoclonal Lambda protein without detectable associated IgG, IgM, or IgA heavy chain. Serum IgM was low at 12.0 mg/dL (reference range 40.0–230.0 mg/dL), serum IgG was also low at 330 mg/dL (reference range 700–1600 mg/dL), and serum IgA was normal at 103.0 mg/dL (reference range 70.0–400.0 mg/dL). The patient also had proteinuria with elevated 24 h urine protein of 2802.5 mg/24 h (reference range 5.0–149.0 mg/24 h). 

Rapid Plasma Reagin (RPR), Hepatitis B surface antigen, Hepatitis C virus antibody, and HIV-1/2 antigen and antibodies (fourth generation) were all non-reactive. The phospholipase A2 receptor antibody panel and Lupus anticoagulant were negative. Complement C3 was normal at 146.0 mg/dL (reference range 90.0–180.0 mg/dL) and Complement C4 was slightly elevated at 49.3 mg/dL (reference range 10.0–40.0 mg/dL).

**Figure 8:**
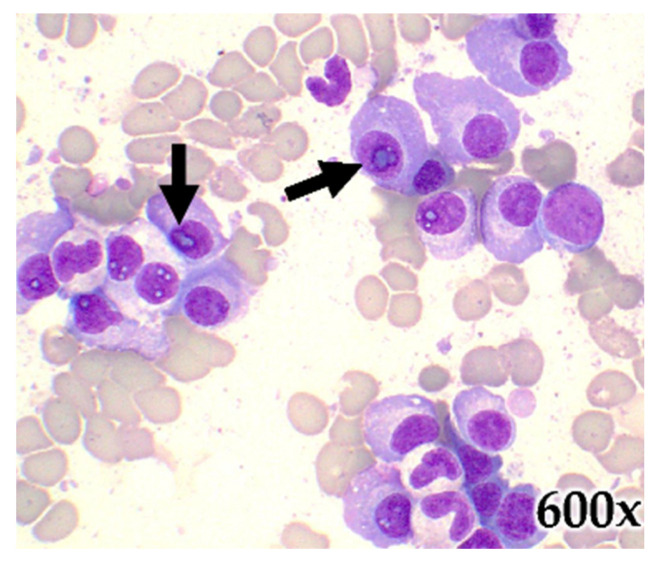
Microscopic image of the bone marrow aspirate. Hematoxylin and eosin staining show increased numbers of plasma cells in clusters, many with intranuclear inclusions (black arrows) (600× objective).

Bone marrow biopsy, aspirate, and peripheral blood smears were performed. The bone marrow aspirate smears revealed increased plasma cells, many with intranuclear inclusions (Dutcher bodies) (Figure 8). The bone marrow aspirated cell block and the EDTA decalcified biopsy sections were approximately 80% cellular. Increased numbers of plasma cells in clusters and sheets were seen. Many of the plasma cells had intranuclear inclusions. Focal deposits of amorphous eosinophilic material were also seen. The immunohistochemical stains for CD138 and MUM-1 were positive, showing increased plasma cells (80% of marrow cellularity). They were positive for cytoplasmic Lambda light chain and BCL-1, and negative for cytoplasmic Kappa light chain (Figure 8). CD3 and CD20 were positive for background T and B lymphocytes. The crystal violet stain was positive for amyloid. 

Bone marrow flow cytometric analysis detected a clonal plasma cell population. A total of 10.8% plasma cells were identified, expressing bright CD38, CD56, and cytoplasmic Lambda light chain with loss of CD19. These results indicated a diagnosis of multiple myeloma and amyloid deposition. 

Chromosome analysis of the bone marrow aspirate revealed a normal female karyotype. Fluorescence in situ hybridization (FISH) analysis of the bone marrow aspirate was performed using a Plasma Cell Myeloma probe panel. Plasma cell enrichment was performed. This study revealed various abnormalities, including: 13q deletion/monosomy 13 (1R1G, 85%; normal < 9.8%), IGH gene rearrangement (1R1G1F, 68%; normal < 11.9%) and t(11;14). Other genetic anomalies associated with high-risk MM were negative.

A final diagnosis of systemic amyloidosis secondary to plasma cell myeloma was made with infiltrative cardiomyopathy and possible diastolic congestive heart failure exacerbation. The patient was discharged on the following medications and asked to follow up with the hematologist oncologist: B complex-vitamin C-folic acid 1 mg, dexamethasone 20 mg, fludrocortisone 0.1 mg, gabapentin 300 mg, and midodrine 10 mg.

## 4. Results and Discussion

### 4.1. Study Designs and Study Population

In our comprehensive review of the literature, a diagnosis of splenic rupture secondary to amyloid light chain (AL) amyloidosis was most often reported as individual case reports [4,7,8,9,10,11,12,13,14,15,16,17,18,19,20,21,22,23]. Other studies included retrospective cohorts [24,25], controlled clinical trials [26], and reviews [27]. Most of these studies were pulled from in-patient hospital data [4,7,8,9,10,11,12,13,14,15,16,17,18,19,20,21,22,23,24,25,26,27]. One case report by Dedi et al. at Leeds General Infirmary, UK, presented the oldest data of the included studies and discussed the case of a 59-year-old woman with AL amyloidosis who suffered a splenic rupture several days following minor trauma [23]. In total, the patients consisted of both men and women between the ages of 43 and 87 [4,7,8,9,10,11,12,13,14,15,16,17,18,19,20,21,22,23,24,25,26,27] (Table 2).

The studies extracted data from various areas and countries, while four studies utilized data from patients in the United States. One study was from Mayo Clinic in Minnesota [8], another was from Geisinger Wyoming Valley Hospital in Pennsylvania [24], one was from Boston University Medical Center in Massachusetts [9], and the last was also from Boston University School of Medicine in Massachusetts [13]. The rest of the studies used data from other countries, including Norway, Korea, China, Switzerland, the UK, Iran, Spain, Mexico, Italy, Taiwan, Japan, Austria, Hong Kong, Slovenia, and Norway [4,7,10,11,12,14,15,16,17,18,19,20,21,22,23,25,26,27]. 

### 4.2. AL Amyloidosis as a Risk Factor for Splenic Rupture

Nontraumatic splenic rupture (NSR) is a rare complication of AL amyloidosis. However, when NSR has been documented, a common culprit seems to be this condition [8]. What seems to still be inconclusive among the literature is what makes it more likely for someone with AL amyloidosis to develop NSR, as well as how to treat this complication when it arises. Worel et al. concluded, at the end of their controlled clinical trial, that treatment of select patients with AL amyloidosis using high-dose melphalan and stem-cell support results in the reversal of amyloid-related disease in a substantial proportion of patients and improved survival [25]. However, they made this claim based on a study with six participants in whom, after 31–52 months of follow up, there was reversal of nephrotic syndrome in those who had renal involvement (n = 3), and one patient had cardiac disease that improved from NYHA class II to class I. They also stated that all participants who received the treatment experienced profound toxicity in response to the medication, and they did not discuss the outcomes of the other disease manifestations that the participants had and whether or not the treatment ameliorated or worsened these other factors [25]. On the contrary, other studies concluded that peripheral stem cell mobilization was a risk factor for the development of NSR [11,13,17,21,26]. Other proposed risk factors for NSR in the setting of AL amyloidosis were factor X deficiency and receiving G-CSF [7,9,11,21]. 

The majority of the case reports described previously healthy patients who presented to the emergency department following sudden onset of abdominal pain, hypotension, oliguria, tachycardia, and other symptoms of hypovolemic shock with no prior diagnosis of AL amyloidosis [4,7,8,9,10,12,15,16,19,20,21,22]. CT scans were diagnostic of splenic rupture in each case, except for one [7]. Each of these patients described no history of recent trauma, except one, who described a minor fall several days prior to the onset of symptoms, suggesting a delayed presentation secondary to initial encapsulation of the bleed [23]. In each of these cases, patients received exploratory laparoscopic surgeries confirming the splenic rupture, with splenectomy following thereafter, except for one case, in which the patient elected to undergo splenic artery embolization since a perisplenic bleed was detected without splenic artery extravasation [13]. This last case concluded that non-operative management should be considered as an alternative to splenectomy; however, this was the only case that involved a patient who had not yet had a splenic rupture, but rather, a perisplenic bleed.

Overall, the majority of these studies concluded that a high degree of suspicion must be used in patients who present with symptoms of hypovolemic shock and abdominal pain without a recent history of trauma or infection, as it may be a rare complication such as NSR secondary to undiagnosed AL amyloidosis [4,7,10,15,17,19,20,21,23,27]. Following diagnosis with a CT scan, an emergent laparotomy and total splenectomy can be lifesaving [4,7,8,9,11,13,16,17,18,27]. 

It has been reported that about 10% to 15% of patients with multiple myeloma may develop AL amyloidosis, while approximately 10% of patients have coexistent AL amyloidosis at diagnosis [17]. There are different hypotheses explaining the possible mechanisms behind NSR due to amyloidosis. The accumulation of amyloid fibrils in the spleen leads to splenomegaly, which renders the spleen fragile and prone to rupture. Although the exact mechanisms leading to NSR in amyloidosis are not fully understood, several factors have been implicated. First, the deposition of amyloid fibrils in the spleen can cause structural changes in the organ, leading to a decrease in its elasticity and fragility. Second, amyloid deposits can cause vascular fragility and rupture of the small vessels within the spleen. Third, the accumulation of amyloid fibrils in the spleen can lead to the formation of nodules or masses, which can exert pressure on adjacent tissues and cause rupture. Finally, the spleen can become enlarged due to the accumulation of amyloid fibrils, making it more susceptible to injury and rupture. Additionally, there are other factors that can contribute to NSR, including hypertension (an increase in the pressure within the spleen) and coagulopathy (which leads to bleeding disorders and can increase the risk of spontaneous bleeding and rupture).

Other studies referred to cases of splenic rupture following autologous stem cell transplantation [11,28]. The exact mechanisms are not well understood, but several factors are believed to contribute to this process: first, the mobilization of stem cells from the bone marrow to the peripheral blood can cause an increase in the size and fragility of the spleen, which can be especially problematic in AL amyloidosis, where the accumulation of amyloid fibrils in the spleen can cause structural changes and increase its fragility; second, the use of granulocyte colony-stimulating factor (G-CSF) to mobilize stem cells can also increase the risk of splenic rupture by increasing white blood cell and platelet counts, which, in turn, contribute to the fragility of the spleen [28,29]; third, the procedure itself can be traumatic, and the insertion of a catheter into the peripheral vein can cause damage to the spleen. To reduce the risk of splenic rupture in such patients undergoing autologous stem cell transplantation, careful monitoring of spleen size can be performed before and after the procedure. Additionally, using alternative mobilization strategies that do not involve the use of G-CSF may help reduce the risk.

### 4.3. Limitations

The biggest limitation currently in the literature is that the vast majority of data available on splenic rupture in the setting of AL amyloidosis comes from case reports of individual patients [4,7,8,9,10,11,12,13,14,15,16,17,18,19,20,21,22,23]. This complication is very rare, and thus, it is difficult to generalize the symptoms, work-up, and treatment suggested in the conclusions of each case report. Even in the retrospective cohort [24], systematic review [26,27], and controlled clinical trial [25] the sample sizes were still extremely small, with the largest sample size being 69 subjects [24], meaning the results could have easily been skewed by outliers. Additionally, even when there was a larger sample size involved, all of the data typically came from the same hospital, which would result in low external validation, except for the study performed by Chiu et al., which gathered information from multiple hospitals [24]. Another limitation is recall bias in patients; they might not have remembered experiencing recent trauma, which could lead to splenic rupture since this presentation can be delayed and can also result from minor trauma. This could mean that splenic rupture could have occurred whether the patient had AL amyloidosis or not. Since they were generally unlisted, it is also difficult to determine whether these patients had other comorbidities that could have contributed to the splenic rupture, and those who did have listed comorbidities tended to have several other health problems [8,14,17]. One case report by Li et al. did not have access to original labs or images since they were carried out at another hospital, which means they could not interpret the information presented [12]. Additionally, many of the cases presented were very short and may not have presented all of the pertinent details [9,15,17,18]. Another case report by Bruserud et al. determined that early recognition of hemorrhagic shock and prompt action restore intravascular volume and oxygen saturation and are lifesaving; however, the patient was still in the hospital recovering at the time of the writing, and it is unknown whether or not that patient was able to go home [4]. Lastly, these case reports and studies came from several different countries which, while good for generalizability, could have contributed to differences in the types of intervention performed at the time of patient presentation due to differences in the quality of healthcare. 

## 5. Conclusions

Splenic rupture secondary to AL amyloidosis is an extremely rare complication. The literature on this topic is scarce, particularly within the United States. The available literature consists mainly of case reports, which in itself provides many limitations when it comes to suggestions on the workup, diagnosis, and treatment of this complication. It would be beneficial to the medical community for any new research to be presented on the topic so that more definitive methods can be established. Additionally, our study emphasizes the importance of including amyloidosis in the differential diagnosis of splenic rupture, and further assessing the causes of amyloidosis to not miss plasma cell myeloma (as such a presentation of plasma cell myeloma is extremely rare and unusual, but worth keeping in mind). 

## Figures and Tables

**Table 1:** Relevant laboratory results of the patient on first admission (11 September 2022).

Blood Test	Patient Value	Reference Range
White blood cell (WBC) count	11.92 × 10^3^/μL	4.8–10.8 × 10^3^/μL
Segmented neutrophils	55.0%	42–75%
Absolute neutrophil count	7.33 × 10^3^/μL	1.8–7.2 × 10^3^/μL
Red blood cell (RBC) count	3.66 × 10^6^/μL	3.93–5.22 × 10^6^/μL
Hemoglobin	11.7 g/dL	12.0–16.0 g/dL
Hematocrit	34.4%	37.0–47.0%
MCV	94.0 fL	79.0–92.2 fL
MCH	32.0 pg	25.6–32.2 pg
MCHC	34.0 g/dL	32.0–36.0 g/dL
Platelet count	323 × 10^3^/uL	150–450 × 10^3^/uL
SARS-CoV-2	NOT DETECTED	NOT DETECTED
HgbA1C	6.5%	4.2–5.6%
C-Reactive Protein	14.7 mg/L	0.0–3.0 mg/L
Blood smear	Large platelets seen	-

**Table 2:** Summary of the studies included in our comprehensive review.

Author (Year)	Country	Design/ Participants	Main Findings	Strengths/Weaknesses	Comments
Bruserud et al., 2021 [4]	Norway	Case report, 67-year-old male	Early recognition of hemorrhagic shock and prompt action restored intravascular volume and SpO_2_ and were lifesaving	Weaknesses: 1 patient was still in hospital receiving treatment when this was written	NSR 2/2 AL amyloidosis patient received 3 courses of chemo with no change in serum lambda levels, treated with daratumumab (which stabilized lambda levels), exploratory laparotomy, and splenectomy
Chiu et al., 2022 [24]	USA	Retrospective cohort, 69 patients with spleen amyloidosis from 2008–2020	Atraumatic splenic rupture was the most common reason for splenectomy in AL cases	Strength: larger sample size, good generalizability since data came from multiple hospitals, had original copies of all data	Only 2 patients had a prior amyloid Dx. Found AL-lambda had diffuse pattern of amyloid deposition with extensive architectural effacement and was more frequently associated with splenic rupture
Lessi et al., 2015 [7]	Italy	Case report, 50-year-old woman	Physicians should monitor for NSR when peripheral stem cell mobilization with G-CSF or both G-CSF and plerixafor is performed in patients with AL amyloidosis due to risk of splenomegaly	Weakness: 1 patient	Previously healthy patient, Dx with cardiac AL amyloidosis, underwent peripheral stem cell mobilization and G-CSF; US showed NSR, patient underwent splenectomy and recovered
Buzalewski et al., 2019 [8]	USA	Case report, 68-year-old male	Most common cause of atraumatic splenic rupture is hematologic processes; amyloidosis is rare	Weakness: 1 patient, multiple comorbidities, multiple prior treatments, multiple hematologic diagnoses	CT on admission showed splenomegaly and splenic hematoma, treated with exploratory laparotomy and splenectomy; multiple post op complications; Dx with AL amyloidosis and MM
Oran et al., 2003 [9]	USA	Case report on 4 patients (1) 52-year-old male (2) 46-year-old female (3) 59-year-old male (4) 56-year-old female	G-CSF may play a role in the development of AL amyloidosis, factor X deficiency may contribute; splenectomy is safe in these patients. There is little rationale for splenic salvage	Weakness: small sample size, short cases may not provide all necessary details	Splenic rupture was manifestation of disease in 1 case, 3 cases had splenic rupture during or after HDM/SCT, 2 had factor X deficiency, all 4 had splenectomy that was uncomplicated
Chang et al., 2017 [10]	Taiwan	Case report, 87-year-old male	Most cases of splenic amyloidosis are global, but it was local in this case. Clinicians should be aware that splenic amyloidosis can lead to hyposplenism with infection and increased risk of NSR	Weakness: 1 patient	Dx with splenic cyst, failed 1st line therapy. Microscopic eval of abscess showed light chains. Dx isolated splenic AL amyloidosis
Sato et al., 2018 [11]	Japan	Case report, 50-year-old male	Special attention is required for amyloidosis patients undergoing auto-SCT even when GCSF is not administered. Atraumatic splenic rupture is rare but is associated with high mortality rate. Amyloid proteins in spleen cause capsule to become fragile	Weakness: 1 patient	Dx MM complicated AL amyloidosis. Splenic rupture complicated during autologous stem cell transplantation (SCT) in absence of GCSF administration, which has previously been linked to amyloidosis splenic rupture. Patient underwent emergent open splenectomy
Li et al., 2019 [12]	China	Case report, 45-year-old male	Dx of this disease took 12 months, suggesting a challenge to Dx. Congo red stain is the gold standard. There are many ways to detect and treat AL amyloidosis. Chemo requires close monitoring	Weakness: 1 patient, and many test results were performed at a prior hospital and the original images or results could not be obtained in those cases	Dx with NSR and hemorrhagic shock, patient had multiorgan involvement and was treated with RPCD regimen. Stable disease state but gradual drug tolerance and insufficient therapeutic response
Bosch et al., 2015 [13]	USA	Case report, 44-year-old female	Splenic artery embolization can be a successful management strategy for the treatment of NSR in AL amyloidosis	Weakness: 1 patient, and cannot say for sure that the spleen would have ruptured without the embolization	The patient was treated with induction therapy with bortezomib, and dexamethasone followed by HDM/SCT, admitted 7 days later. CT showed perisplenic bleeding. Splenic artery angiography showed no active extravasation. Underwent distal splenic artery embolization and no further hemodynamic instability
Worel et al., 2006 [25]	Austria	Controlled clinical trial, 6 patients between 43 and 59 years old, previously Dx with AL amyloidosis, previously treated with rhG-CSF alone or treated with cyclophosphamide and rhG-CSF	Treatment of select patients with AL amyloidosis with high-dose melphalan and stem-cell support results in reversal of amyloid-related disease and improved survival	Weakness: risk for confounding, and researcher’s conclusion is too generalized	Of all the patients, 1 patient died from sepsis after stem-cell mobilization, 5 had high-dose melphalan and had severe toxicity, 1 died from GI perf, 1 had hyperfibrinolysis and spontaneous splenic rupture, 1 had severe bleeding, 3 needed hemodialysis, and 1 had a renal transplant
Chau et al., 2008 [14]	Japan	Case report, 74-year-old female	Splenomegaly not needed for NSR. Emergency laparoscopic splenectomy is necessary	Weakness: 1 patient, extensive comorbidities	Dx with AL amyloidosis on peritoneal dialysis, patient underwent laparoscopy, which showed NSR with normal-size spleen
Choudhuri et al., 2008 [15]	UK	Case report, 59-year-old male	Splenic peliosis is an extremely rare manifestation of MM, urgent operative intervention is required	Weakness: 1 patient, very short case report, and other necessary information may be missing	Dx with MM which was asymptomatic; 6 years later, patient experienced renal impairment, and then, had NSR, emergency splenectomy. Patient had splenic peliosis
Magnoli et al., 2013 [16]	Italy	Case report, 49-year-old woman	Very aggressive behavior of MM is related to peculiar molecular features with cell cycle progression and microenvironment interactions	Weakness: 1 patient	NSR on CT. Splenectomy complicated by consumption coagulopathy. Patient died from DIC and multiple organ dysfunction syndrome. Performed path exam of spleen and found MM
Perrone et al., 2019 [17]	Italy	Case report, 64-year-old male	AL amyloidosis can evolve from MM. Survival improvement in treatment of MM due to new therapies increases probability of developing amyloidosis. Clinicians should control patients for amyloid-induced organ damage	Weakness: 1 patient, several comorbidities, several treatments provided from Dx to death, possible confounding, and bias	Dx of IgG lambda MM, stem cell transplant, NSR 2/2 amyloidosis, treated with exploratory laparotomy and splenectomy
Renzulli et al., 2009 [26]	Switzerland	Review, 15 women and 16 men with mean age of 53 with atraumatic splenic rupture; 25 had AL amyloidosis, 4 had secondary amyloidosis, 2 had unspecified amyloidosis	NSR in amyloidosis has a high 30-day mortality rate and occurs mostly in those with undiagnosed amyloidosis. Identified splenomegaly, coagulation abnormalities, and autologous stem cell transplant as predisposing factors	Strength: review of multiple sources, good generalizability of findings Weakness: small sample size	Of 31 patients with NSR in amyloidosis, 79% had NSR as initial manifestation, 8% were affected with MM. Splenic rupture 30-day mortality rate = 26%
Roh and Huh 2015 [18]	Korea	Case report, 53-year-old male	Identified amyloidosis with deposition of pink/amorphous material microscopically and apple-green birefringence via Congo red stain	Weakness: 1 patient, very short case report	Dx MM, CT showed nontraumatic splenic rupture
Elvy et al., 2011 [27]	UK	Retrospective cohort, 7 patients from the same hospital over 6 years	CT facilitates Dx. Early total splenectomy is needed. High index of suspicion should be maintained by ER physicians	Weakness: same hospital so low generalizability, and small sample size	All patients required splenectomy; 1 had amyloidosis
Shobeiri et al., 2013 [19]	Iran	Case report, 61-year-old male	In patients with abdominal pain and hypotension, NSR should be suspected in those without trauma or infection Hx	Weakness: 1 patient	Ultrasound showed NSR; performed laparotomy and found hemoperitoneum 2/2 NSR. Path examination of spleen showed AL amyloidosis
Garcia et al., 2010 [20]	Mexico	Case report, 46-year-old male	In patients with abdominal pain and hypotension, NSR should be suspected in those without trauma or infection Hx	Weakness: 1 patient	Performed laparotomy and found hemoperitoneum 2/2 NSR. Performed splenectomy, and path report showed AL amyloidosis
Fernandez de Larrea et al., 2008 [21]	Spain	Case report, 51-year-old woman	Only a few cases of splenic rupture from stem cells have been reported. NSR is a medical emergency that needs rapid Dx, especially in those with factor X deficiency or undergoing G-CSF	Weakness: 1 patient	Found AL amyloidosis on renal biopsy. Patient had NSR 24 h after infusion of peripheral blood stem cells and died from multiorgan failure
Dedi et al., 2001 [23]	UK	Case report, 59-year-old woman	Splenic rupture should be considered in the Dx of hemoperitoneum complicating peritoneal dialysis, especially in those with amyloidosis. Injury may precede clinical presentation	Weakness: 1 patient, stated that the bleed “appeared” to be from minor trauma, but that cannot be known	Patient on dialysis for end-stage renal failure 2/2 AL amyloidosis; had a minor fall downstairs and admitted 4 days later. Died from numerous complications 6 weeks later. Found amyloidosis in spleen and subcapsular splenic bleed
Skok et al., 2009 [22]	Slovenia	Case report, 52-year-old male	Splenomegaly is a risk factor for splenic rupture in patients with AL amyloidosis	Weakness: 1 patient	Patient previously treated for undefined hepatic disease and anemia, confirmed posthumously to be AL amyloidosis. Died from NSR

## Data Availability

Not applicable.
